# Utilization and associated factors of reproductive health services among 20–39-year-old women in rural China: a cross-sectional study

**DOI:** 10.1186/s12978-021-01182-z

**Published:** 2021-06-27

**Authors:** Jing Yue, Yang Luo, Chen Xu, Si Qin, Yanting Meng, Ling Fan, Min Nie

**Affiliations:** Xiang Ya Nursing School, Central South University, Changsha, China

**Keywords:** Reproductive health, Service, Utilization, Rural, China

## Abstract

**Background:**

The use of reproductive health (RH) services is important to promote RH. However, little is known about RH services in rural areas, especially in low- and middle-income countries. China is the most populous country in the world, and 40.4% of its population is rural. Our study determined the utilization of and factors associated with RH services in rural China.

**Methods:**

A cross-sectional study of 978 20- to 39-year-old women was performed in four villages of four cities in Hunan Province. A researcher-created structured questionnaire was used to collect the data. The data were entered into EpiData v3.0 and analysed using SPSS v18.0. Statistical significance was defined as a two-sided *P*-value of less than 0.05. Descriptive statistics were used to examine the socio-demographic factors and the use of RH services by the sample population. Chi-square tests were used to assess associations between categorical variables. Logistic regression analyses were performed to examine factors that correlated with the use of RH services.

**Results:**

The top three services used were antenatal examinations (90.2%), postpartum visits (73.0%) and free folic acid supplements (71.6%). Age, monthly household income, employment, spousal education level, and artificial abortion history were associated with RH service utilization (*P* < 0.05). The most desired RH service was cervical/breast cancer prevention services (58.9%). The most preferred method participants used to obtain information on RH services was the internet.

**Conclusions:**

The utilization rate for RH services in rural China needs improvement. Future efforts should target high-risk populations of women by providing them with RH-related information and cultivating positive attitudes towards RH services.

**Supplementary Information:**

The online version contains supplementary material available at 10.1186/s12978-021-01182-z.

## Plain language summary

Reproductive health (RH) services are recognized worldwide as a cost-effective strategy to promote RH. However, little is known about RH services in rural areas, especially in low- and middle-income countries. China is the most populous country in the world, and 40.4% of its population is rural. Due to the implementation of China’s universal two-child policy, women between 20 and 39 years old are likely to experience more RH problems related to childbirth. Our study investigated the utilization of and factors associated with RH services in rural China. The top three services used among the 978 participants were antenatal examinations (90.2%), postpartum visits (73.0%) and free folic acid supplements (71.6%). Age, monthly household income, employment, spousal education level, and artificial abortion history were associated with RH service utilization. The participants most desired services pertained to the prevention of cervical/breast cancer, and the most preferred methods used to obtain information related to RH services was the internet.

In conclusion, the utilization of RH services requires improvement, and future efforts should target high-risk populations of women by providing them with RH-related information and cultivating positive attitudes towards RH services.

**Supplementary Information:**

The online version contains supplementary material available at 10.1186/s12978-021-01182-z.

## Background

Reproductive health (RH) refers to a state of complete physical, mental, and social well-being (and not merely the absence of disease or infirmity) in all matters relating to the reproductive system and its functions and processes [1]. RH is closely related to human reproduction and social development [2, 3]. RH services refer to the medical and health services provided for the realization of reproductive health, including sex education, sexual and reproductive health consultation, contraceptives, and medical and health services [4]. The United Nations (UN) Sustainable Development Goals (SDGs) include ensuring universal access to sexual and reproductive health care services by 2030 [5]. Therefore, it is necessary to identify the uses and obstacles of RH services.

A total of 3.4 billion people live in rural areas worldwide, representing 44.0% of the global population [6]. China is the most populous country in the world, and the rural population accounts for 40.4% of its total population. One study in rural China showed that 53% of the respondents had a low RH knowledge score, and 39% of respondents with gynecopathy-related discomfort did not seek professional help [7]. Since the implementation of the universal two-child policy in 2016, every couple in China has been encouraged to have two children [8]. The number of women between 20 and 39 years old choosing to give birth increased rapidly in a short time. RH problems related to childbirth have also become more prominent [9, 10]. Women between 20 and 39 years old are likely to experience more RH problems related to childbirth. Therefore, identifying the uses of RH services and the factors influencing their use are urgently needed.

Although some studies have examined the use of RH services in Chinese women, there are still some limitations of the available studies [11–15]. Some of the problems we observed in our review were that studies were conducted before the implementation of the universal two-child policy, obtained samples from medical units, and did not analyse associated factors very well. Our study targeted large-scale community women aged 20-39 years to investigate the utilization and associated factors of RH services in rural areas after implementation of the universal two-child policy. We also determined the desired RH services and preferred methods of obtaining RH-related information in rural women. These findings will be useful for the implementation of policies and strategies that promote the use of RH services in rural areas.

## Methods

### Study design and sampling

This cross-sectional study was performed in Hunan Province in cooperation with the Women's Federation of the Hunan Provincial Government between May 30 and September 16, 2019. Hunan Province is a major province in central China with a rural population of 30.3 million. The birth rate in 2018 was the highest of the three regions of China (i.e., western, central, and eastern China) at 1.2% compared with 1.0% in eastern China and 1.1% in western China [16]. We selected four counties in the eastern (Loudi), western (Huaihua), southern (Chenzhou), and northern (Changde) regions of Hunan Province that were representative of each region based on economic and demographic factors. We then selected one city in each county. Multistage cluster sampling was used to recruit participants from the four selected cities. We randomly selected one township per city and one village from each township for a total of four villages.

### Sample size

We determined the sample size using a random sampling formula. A study of the floating population estimated that the utilization rate of RH services in China was approximately 40.0% [17], and we chose an approximate value of 40.0% to calculate the sample size. The following formula was used to calculate the sample size: **n = μ**^**2**^_**α**_** × P(1-P)/δ**^**2**^, where α = 0.05, μ_α_ = 1.96, P was the overall rate of RH service use, and δ = 3.3%. The calculated sample size was 847. To reduce sampling error and increase the study power, we made a rough estimation by arbitrarily multiplying the calculated sample size by 15%, which led to a sample size of 974. Ultimately, 980 women who met the requirements were recruited, and 978 (99.8%) participants completed the questionnaire as requested.

### Participants

Dynamic population monitoring data (marriage, pregnancy, birth, death and migration), which were collected and updated regularly by the health department from Hunan Province, revealed that the fertility rate of women over 40 years was less than 2.0% [18]. Therefore, women aged 20–39 years were selected in our study to investigate the utilization of and factors associated with RH services. The inclusion criteria were: (1) females between 20 and 39 years old, (2) females with residence registration (hukou) in rural Hunan or who had lived in the investigated area for more than 6 months, and (3) females who provided oral informed consent. The exclusion criteria were cognitive impairment and the inability to complete the questionnaire independently. All participants were told that their involvement was voluntary and that they could withdraw at any time.

### Measurements and data collection

The Women’s Reproductive Health Questionnaire (WRH-Q) was developed by the researchers after an extensive literature review and revised by a panel of 10 experts from Central South University, affiliated hospitals, and the Hunan Women's Federation (see Additional file 1). We recruited women via direct door-to-door visits. When three door-to-door visits failed (because the subjects were out of the house or refused to participate), the household was abandoned and replaced with an alternate household in sequence.

The Women's Federation of the Hunan Provincial Government trained community health workers who were familiar with the locality as investigators. The training content included the content and purpose of the current study and the process for collecting the questionnaires. Well-trained investigators performed interviews with eligible subjects and explained the study purpose and procedures. The anonymous questionnaires were completed by the subjects themselves or with the help of the investigators if the subjects had limited literacy. Personnel from the local maternal and child health facility acted as auditors and were responsible for questionnaire collection and quality control. Auditors assessed the completeness and accuracy of each questionnaire on the spot and asked for additional information or corrections if any omissions or errors were found.

### Socio-demographic and gestation data

We collected information on each participant’s age, marital status, employment, monthly household income, education level, spouse’s education level, sexually active status, artificial abortion history, pregnancy history, and delivery history.

### Utilization of RH services

RH services for women of childbearing age in China primarily include consultations, premarital check-ups, preconception care, antenatal examinations, postpartum visits, and general gynaecological examinations [16]. The questionnaire collected the following information: (1) utilization of RH counselling, in which participants answered questions about whether they received contraception and/or pregnancy counselling; (2) utilization of RH-related examinations or services, in which participants answered questions on whether they received free folic acid supplements; free AIDS, syphilis and hepatitis B testing; free antenatal examinations; free preconception care; and free postpartum visits; and (3) utilization of RH screening, in which participants answered questions regarding whether they received cervical and breast cancer screening. Participants who used all RH counselling/RH screening services and four or more RH-related examinations/services were categorized as participants who sufficiently utilized RH services.

### Needs for RH services

The structured questionnaire also asked about the women’s needs for RH services. (1) What type of RH services do you desire to receive? (Options: prevention of cervical/breast cancer, child health care, reproductive tract infection/sexually transmitted disease treatment, psychological health care, pregnancy/prenatal care, contraception, menstrual hygiene, and others). (2) What method do you prefer to use to obtain RH service-related information? (Options: medical staff, internet, WeChat/microblogs, radio and television, friends and family, books/newspapers/magazines, and brochures).

### Data analysis

All data were assessed for errors and double-entered independently into EpiData (Version 3.0., The Epidata Association, Odense, Denmark). Data were analysed using SPSS (Version 18.0., Chicago: SPSS Inc.). Descriptive statistics were used to examine the socio-demographic factors and status of the RH services used by the participants. Differences in the use of RH services between different groups (e.g., age, employment, monthly household income, education level and spouse’s education level, and artificial abortion history) were computed using the chi-square test. Logistic regression analyses were performed to examine factors that correlated with the use of RH services. Statistical significance was defined as a two-sided *P*-value less than 0.05. If greater than 20% of the items had missing values, the questionnaire was excluded as invalid.

### Ethical considerations

The Ethics Committee of Xiang Ya Nursing School, Central South University approved the study (approval number: 2019005). Written consent was obtained from all respondents before the interviews.

## Results

### Participants’ socio-demographic and gestational characteristics

Among the 980 women who were recruited in this study, 978 completed the questionnaire as requested (response rate: 99.8%). Of the 978 women, most of the respondents were married (86.5%), and their average age was 30.2 years (SD = 4.9). Of the 850 women who were sexually active, 820 had a history of pregnancy (96.5%). Among the 820 women with a history of pregnancy, 257 had a history of artificial abortion (31.4%), and 811 women had a history of delivery (98.9%) (Table 1).

**Table 1:** Socio-demographic and gestational characteristics (N = 978)

Variables	Categories	Frequencies	Percentages
Age (years)	19–25	186	19.0
	26–32	435	44.5
	33–39	357	36.5
Marital status	Unmarried	132	13.5
	Married	846	86.5
Employment	Unemployed	347	35.5
	Employed	631	64.5
Monthly household income (^*^USD)	< 771	625	63.9
	771–1543	322	32.9
	> 1543	31	3.2
Education level	Primary school or less	53	5.4
	Junior high school	388	39.7
	Senior high school	295	30.2
	College or more	242	24.7
Spouse’s education level (N = 846)	Primary school or less	41	4.8
	Junior high school	332	39.3
	Senior high school	293	34.6
	College or more	180	21.3
Sexually active	No	128	13.1
	Yes	850	86.9
Pregnancy history (N = 850)	No	30	3.5
	Yes	820	96.5
Artificial abortion history (N = 820)	No	563	68.6
	Yes	257	31.4
Delivery history (N = 820)	No	9	1.1
	Yes	811	98.9

^*^1 USD≈6.5 RMB (January 24, 2021)

### Utilization of RH services

Of the 850 women who were sexually active, 53.6% received contraception counselling. Among the 820 women who had given birth, 27.5% received free preconception care, and 90.2% received free antenatal examinations. Of the 978 women, approximately one-third received cervical and breast cancer screening (Table 2).

**Table 2:** Utilization of RH services (N = 978)

Variables	Frequencies	Percentages
*RH counselling (n* = *850)*		
Contraception counselling	456	53.6
Pregnancy counselling	303	35.7
*RH screening*		
Cervical cancer screening	359	36.7
Breast cancer screening	333	34.0
*RH examinations/services (n* = *820)*		
Free folic acid supplement	587	71.6
Free AIDS, syphilis and hepatitis B testing	389	47.5
Free antenatal examination	740	90.2
Free preconception care	225	27.5
Free postpartum visit	599	73.0

### Factors associated with the use of RH services

Table 3 presents the results of the univariate analysis of differences in the use of RH services in different groups. Statistically significant differences (*P* < 0.05) in the use of RH screening and RH examinations/services were noted based on age, employment, monthly household income and spouse education level. There were statistically significant differences (*P* < 0.05) in the use of RH counselling based on employment and artificial abortion history.

**Table 3:** Chi-square analysis of differences in the use of RH services in different groups

Measure		RH counselling (N = 850)				RH screening (N = 978)				RH-related examinations/services (N = 820)				
		Insufficiency	Sufficiency	χ^2^	*P-*Value	Insufficiency	Sufficiency	χ^2^	*P-*Value	Insufficiency	Sufficiency	χ^2^	*P-*Value	
*Age (years)*				1.149	0.284			15.985	** < 0.001**			6.373	**0.012**	
19–29		354 (77.2)	104 (22.8)			424 (80.5)	103 (19.5)			161 (36.4)	281 (63.6)			
30–39		313 (79.8)	79 (20.2)			244 (54.0)	207 (46.0)			174 (45.9)	204 (54.1)			
*Employment*				4.971	**0.026**			19.997	** < 0.001**			6.981	**0.008**	
Yes		426 (77.7)	122 (22.3)			387 (61.3)	244 (38.7)			268 (50.6)	261 (49.4)			
No		255 (84.3)	47 (15.7)			296 (85.3)	51 (14.7)			113 (38.8)	178 (61.2)			
Monthly household income (^*^USD)				1.886	0.389			13.118	**0.001**			12.908	**0.002**	
< 771		435 (80.1)	108 (19.9)			449 (71.8)	176 (28.2)			249 (47.5)	275 (52.5)			
771–1543		218 (78.0)	62 (22.0)			206 (64.0)	116 (36.0)			96 (35.5)	174 (64.5)			
> 1543		20 (74.4)	7 (25.6)			21 (66.7)	10 (33.3)			8 (29.1)	18 (70.9)			
*Education level*				2.527	0.283			16.292	** < 0.001**			17.872	** < 0.001**	
Junior high school or less		201 (79.8)	51 (20.2)			187 (64.6)	102 (35.4)			121 (49.6)	122 (50.4)			
Senior high school		194 (80.3)	47 (19.7)			180 (64.7)	98 (35.3)			103 (44.3)	130 (55.7)			
College or more		271 (76.0)	86 (24.0)			301 (73.2)	110 (26.8)			106 (30.7)	238 (69.3)			
*Spouse’s education level*				1.509	0.470			17.849	** < 0.001**			20.918	** < 0.001**	
Junior high school or less		238 (80.6)	57 (19.4)			220 (64.9)	119 (35.1)			151 (53.0)	134 (47.0)			
Senior high school		223 (78.5)	61 (21.5)			192 (58.6)	135 (41.4)			113 (41.3)	161 (58.7)			
College or more		209 (77.2)	62 (22.8)			160 (51.4)	152 (48.6)			84 (32.2)	177 (67.8)			
*Artificial abortion history*				15.614	** < 0.001**			12.769	** < 0.001**			24.988	** < 0.001**	
Yes		183 (68.5)	84 (31.5)			196 (64.0)	111 (36.0)			136 (53.1)	121 (46.9)			
No		497 (85.2)	86 (14.8)			364 (54.2)	307 (45.8)			87 (33.9)	170 (66.1)			

Table 4 presents the results of the logistic regression analysis on the relationship between the associated factors and RH service utilization. Compared with women aged 19–29, 30–39-year-old women were more likely to use RH screening (OR = 1.985, 95% CI: 1.555–2.533) but were less likely to use RH-related examinations/services (OR = 0.710, 95% CI: 0.513–0.983). Compared with women who were unemployed, employed women were more likely to use RH counselling (OR = 1.678, 95% CI: 1.138–2.476), RH screening (OR = 2.060, 95% CI: 1.504–2.821), and RH-related examinations/services (OR = 1.510, 95% CI: 1.035–2.205). Compared with women whose monthly household income was less than 771 USD, women with monthly household income between 771 and 1543 USD were more likely to use RH screening (OR = 1.681, 95% CI: 1.320–2.142). Compared with women whose spouse’s education level was junior high school or less, women with a higher spouse’s education level were more likely to use RH screening (OR = 1.335, 95% CI: 1.009–1.765; OR = 1.649, 95% CI: 1.233–2.204, respectively) and RH-related examinations/services (OR = 1.675, 95% CI: 1.115–2.515; OR = 1.976, 95% CI: 1.313–2.973, respectively). Women who had a history of artificial abortion were more likely to use RH counselling (OR = 2.743, 95% CI: 2.081–3.615).

**Table 4:** Logistic regression analysis for factors associated with the use of RH services

Measure	RH counselling (N = 850)			RH screening (N = 978)			RH-related examinations/services (N = 820)		
	Coefficient	OR (95% CI)	*P-*Value	Coefficient	OR (95% CI)	*P-*Value	Coefficient	OR (95% CI)	*P-*Value
*Age (years)*									
19–29	1			1			1		
30–39	− 0.256	0.774 (0.581–1.032)	0.081	0.686	1.985 (1.555–2.533)	** < 0.001**	–0.342	0.710 (0.513–0.983)	**0.039**
*Employment*									
No	1			1			1		
Yes	0.518	1.678 (1.138–2.476)	**0.009**	0.723	2.060 (1.504–2.821)	** < 0.001**	0.412	1.510 (1.035–2.205)	**0.033**
Monthly household income (^*^USD)									
< 771	1			1			1		
771–1543	0.030	1.030 (0.763–1.391)	0.845	0.520	1.681 (1.320–2.142)	** < 0.001**	0.344	1.411 (0.988–2.015)	0.058
> 1543	0.095	1.099 (0.620–1.949)	0.747	0.169	1.184 (0.735–1.907)	0.487	0.627	1.872 (0.979–3.579)	0.058
*Education level*									
Junior high school or less	1			1			1		
Senior high school	− 0.111	0.895 (0.613–1.306)	0.564	0.022	1.022 (0.751–1.391)	0.890	0.008	1.008 (0.657–1.547)	0.971
College or more	0.213	1.237 (0.780–1.962)	0.365	0.115	1.122 (0.763–1.650)	0.557	0.201	1.222 (0.720–2.073)	0.457
*Spouse’s education level*									
Junior high school or less	1			1			1		
Senior high school	0.050	1.051 (0.720–1.536)	0.796	0.289	1.335 (1.009–1.765)	**0.043**	0.516	1.675 (1.115–2.515)	**0.013**
College or more	− 0.019	0.981 (0.619–1.556)	0.936	0.500	1.649 (1.233–2.204)	**0.001**	0.681	1.976 (1.313–2.973)	**0.001**
*Artificial abortion history*									
No	1			1			1		
Yes	1.009	2.743 (2.081–3.615)	** < 0.001**	0.147	1.159 (0.716–1.875)	0.549	0.269	1.308 (0.823–2.081)	0.256

*OR* odds ratio, *95% CI* 95% confidence interval

### Participants most desired RH services

**Fig. 1:**
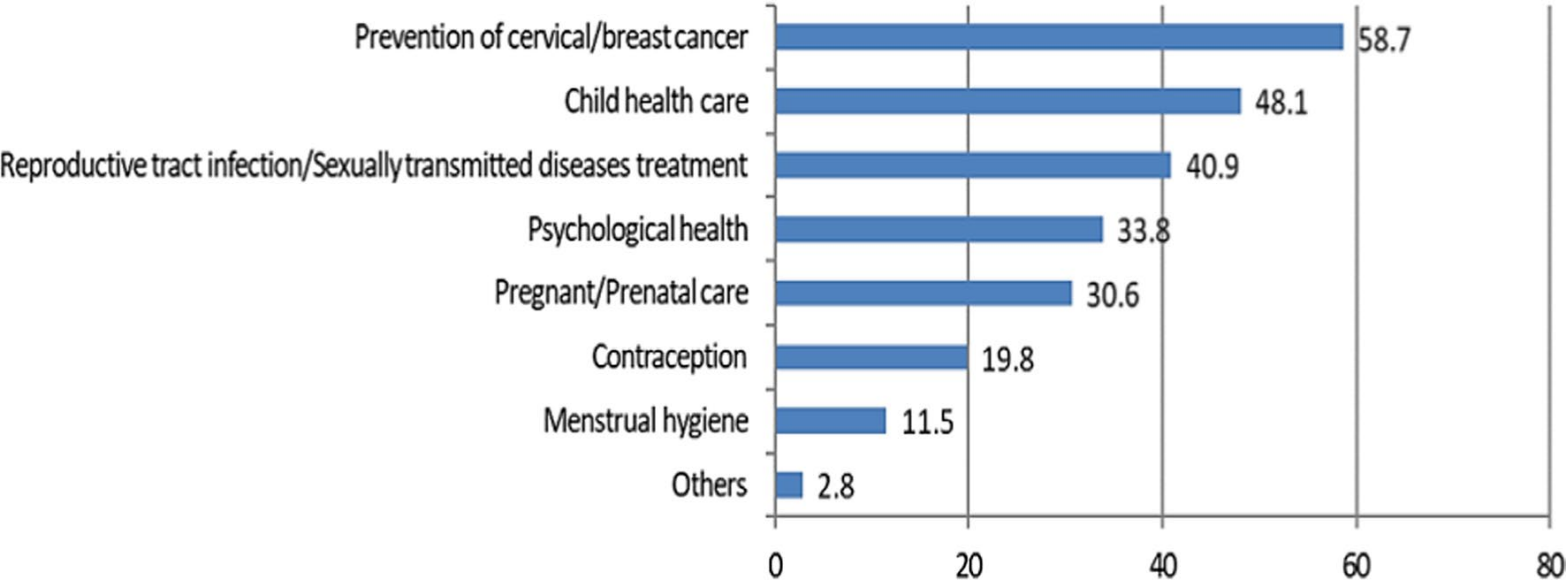
The most desired RH services (reported as percentage of participants)

The top three RH services participants most desired were prevention of cervical/breast cancer (58.9%), child health care (48.2%), and reproductive tract infection/sexually transmitted disease treatment (41.1%) (Fig. 1).

### The most preferred methods to obtain RH service-related information

**Fig. 2:**
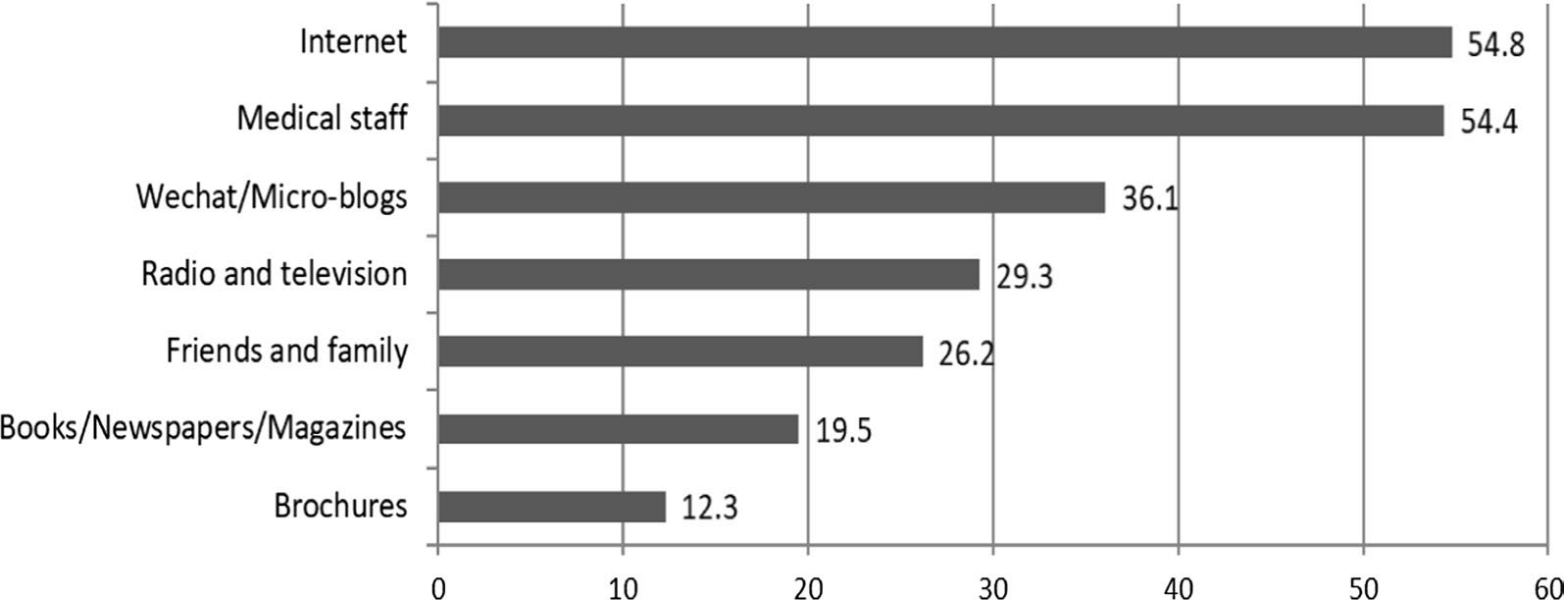
The most preferred methods of obtaining RH service-related information (expressed as percentage of participants)

The top three methods participants preferred to use to obtain RH service-related information were the internet (54.8%), medical staff (54.4%), and WeChat/microblogs (36.1%) (Fig. 2).

## Discussion

The total proportion of females receiving RH services in rural China needs improvement. Age, monthly household income, employment, spouse education level, and artificial abortion history were associated with the use of RH services. The RH services women most desired pertained to the prevention of cervical/breast cancer. The most preferred method for participants to obtain information about RH services was the internet.

The most commonly used RH service was antenatal examinations (90.2%) in our study, demonstrating that the policy of promoting free antenatal examinations in China has achieved some success. The Chinese government stipulates that employed women are entitled to antenatal examination leave after pregnancy to give them more access to medical care [19]. However, the use of this service was below the national level of 95.0% [16]. This lower rate of use may be due to the increased proportion of older pregnant women under implementation of the universal two-child policy. These older women had multiple pregnancies and were less concerned about maternal and infant health due to poor risk awareness and successful experiences with previous pregnancies [20, 21].

The second most commonly used RH service was postpartum visits (73.0%). However, the Chinese health authorities state that the proportion of women engaging in postpartum visits after 2015 should be greater than 95.0% [16]. The United States and some developing countries also have barriers to participation in postpartum visits, such as a negative attitude on the part of the providers and a lack of awareness of postpartum care [12, 22]. This finding indicates a need for health care facilities and providers to make concerted efforts to increase knowledge about the importance of postpartum visits and develop interventions for the target population, including education, home visits, incentives, and appointment scheduling initiatives [23, 24].

The third most commonly used RH service was free folic acid supplements (71.6%), which was far below the national level of 95.0% [25]. Although the community provides free folic acid to every woman who desires to become or is already pregnant, most women think they do not need folic acid if they have a healthy pregnancy and think it is inconvenient to obtain folic acid from the community, as noted in a mixed-method study [26]. Therefore, the establishment of a more flexible method of folic acid delivery may facilitate folic acid supplementation. For example, all pregnant women without contraindications can receive a free folic acid supplement at a nearby pharmacy [27].

The least utilized RH service was preconception care (27.5%). The utilization rate was lower than that measured among 12,309 women in Shanghai, China (40%) [13]. The differences among these results may be explained as follows. First, in a study conducted in Shanghai, 56.2% of the participants had a college degree or above. However, in this study, only 24.7% of participants had a college education. Highly educated women might be aware of the importance of preconception care to promote maternal health [28]. Second, we recruited women from communities, whereas the previous study sampled from hospitals when the women presented for their initial prenatal visit. To some extent, we avoided sample bias. Third, China implemented the National Free Preconception Health Examination Project (NFPHEP) in 2011 to provide free preconception care in rural areas [29]. This finding may be attributed to insufficient policy advertisement, but more studies are needed to systematically evaluate the obstacles to NFPHEP implementation in low-income populations in China.

Additionally, we found that women aged 30–39 years were more likely to receive RH screening, which is consistent with previous studies conducted in China [14, 15]. Employed women exhibited better utilization of RH screening and related examinations in this study. The relationship between employment and the utilization of RH services has been insufficiently researched to date. In a study of 850 rural married women in Hainan Province, China, the level of RH knowledge of employed women was greater than that of the general population [30]. Wang [31] found that a high level of RH knowledge was associated with a positive attitude and behaviour. Although further research is necessary, healthcare providers could promote the utilization of RH services for target populations by improving the level of RH knowledge.

Remarkably, women whose spouses had a high education level were more likely to use RH screening and related examinations/services. Husbands can greatly influence decision-making process. An educated husband might be aware of the importance of RH services in promoting women’s health and remind their partner to receive such services [22, 32, 33]. Our results reinforce the importance of targeting families with interventions, not just individual women.

In our study, women who had an artificial abortion history were more likely to use RH counselling. Previous studies on childbearing women found that only education level, region and service quality affected RH counselling use [34, 35]. A possible reason is that, after artificial abortion, doctors may provide women with RH counselling services to avoid the next unintended pregnancy or provide guidance for their next pregnancy [36, 37].

The RH service most desired by participants in this study involved cervical/breast cancer prevention. This finding may be due to the fact that cervical cancer and breast cancer are the most common malignant tumours in women; thus, women hope to receive prevention services [38, 39]. Our study also found that women preferred to access RH service-related information via the internet. The internet is an effective platform for improving health services due to the ease of obtaining information without time and space limitations [40–42]. Other methods of obtaining RH service-related information, such as lectures, brochures, magazines, video and television, should be highlighted as alternative methods to meet the needs of different groups, especially in underdeveloped areas that lack access to the internet.

### Strengths and limitations

This was the first study based on a large sample of childbearing women in central rural China to investigate the utilization of and factors associated with RH services among childbearing women after implementation of the universal two-child policy. We selected four regions to control for sampling errors. Data collectors, individuals involved in quality control, and other personnel were thoroughly trained before the study. All of the participants were recruited from the community, which may have made the sample more representative and the findings more universally applicable.

There are some methodological limitations of this study. First, the data were retrospectively obtained and self-reported, and memory bias and concealment of information were possible. Second, our study only described the current use of and need for RH services given the cross-sectional design, and more intervention studies are needed to promote the use of RH services. Finally, RH is a problem involving multiple generations, i.e., grandmothers and mothers-in-law, and more research is needed to determine whether their attitudes influence women’s decisions about whether to access RH services.

## Conclusion

Although the Chinese government proposed plans to improve the use of RH services in rural areas, we demonstrated that challenges remain regarding the use of such services. The internet may be one of the best and most effective methods for enhancing RH knowledge and improving the use of RH services. Future efforts should target high-risk populations of women who are older, have a low income or low education level, or are unemployed as well as their families by providing them with RH service-related information and cultivating positive attitudes towards RH services.

## Supplementary Information

**Additional file 1.** Women’s Reproductive Health Questionnaire.

## Data Availability

Data are available upon request from the corresponding author.

## Abbreviations

RH: Reproductive health
UN: United Nations
SDG: Sustainable development goals
HPV: Human papillomavirus
NCSPRA: National Cancer Screening Program in Rural Areas
