# Supplementary material for: Utilization and associated factors of reproductive health services among 20–39-year-old women in rural China: a cross-sectional study
Source: Reprod Health. 2021 Jun 27;18:134. doi: 10.1186/s12978-021-01182-z (PMC8237466; doi:10.1186/s12978-021-01182-z)
Supplement: Supplementary file 1 — Additional file 1. Women’s Reproductive Health Questionnaire. [file 12978_2021_1182_MOESM1_ESM.docx]

**Additional file:**

**Women’s Reproductive Health Questionnaire**

Dear Female friend:

Thank you very much for participating in this questionnaire survey about reproductive health (RH) services. The study is carried out by the Reproductive Health Studies team on the Xiangya Nursing School of Central South University.

We want to know about the utilization of and demand for reproductive health services among 20–39-year-old women in rural Hunan. There is no right or wrong answer. And then, you don’t worry about your information being leaked because the questionnaire is anonymous and used for analysis. We hope that you can spare your precious time and fill in this questionnaire according to your actual situation. You have the right not to answer any questions, or wish to exit the field at any time.

We would like to express our heartfelt thanks to you for your cooperation and support. Best wishes!

The Reproductive Health Studies team

Xiangya Nursing School of Central South University

**Part 1 Socio-demographic and Gestational Characteristics**

***Instructions:*** *below are some Socio-demographic and Gestational characteristics about you. Please fill in the blank space or tick the appropriate box.*

1. Region:

2. Your age (years):

3. Your marital status:

□ Married

□ Unmarried

4. Are you be employed?

□ Employed

□ Unemployed

5. Your monthly household income (CNY):

□ < 5000

□ 5000–10000

□ ＞10000

6. Your educational level:

□ Primary school or less

□ Junior high school

□ Senior high school

□ College or more

7. Your spouse's educational level:

□ Primary school or less

□ Junior high school

□ Senior high school

□ College or more

8. Have you ever had sex?

□ No (please jump to the question 20)

□ Yes

9. Have you ever been pregnant?

□ No

□ Yes

10. Have you ever had an artificial abortion?

□ No

□ Yes

11. Have you ever given birth?

□ No (please jump to the question 20)

□ Yes

**Part 2 Utilization of RH services**

***Instructions:*** *below are some* *statements concerning reproductive health services. Please indicate whether you have accepted the relevant services by ticking the appropriate box.*

**RH counseling**

12. Do/did you get contraception counseling?

□ No

□ Yes

13. Do/did you get pregnancy counseling?

□ No

□ Yes

**RH-related examinations or services**

|  | Yes | No |
| --- | --- | --- |
| 14. Free folic acid supplements |  |  |
| 15. Free AIDS, syphilis and hepatitis B testing |  |  |
| 16. Free antenatal examinations |  |  |
| 17. Free preconception care |  |  |
| 18. Free postpartum visits |  |  |
| 19. Cervical cancer screening |  |  |
| 20. Breast cancer screening |  |  |

**Part 3 Demand for RH services**

***Instructions:*** *below are some statements concerning the needs for reproductive health services. Please indicate your opinion by ticking the appropriate box.*

21. What type of RH services do you desire to get? ([multiple](javascript:;)-[choice](javascript:;), a maximum of three choices)

□ Prevention of cervical/breast cancer

□ Child health care

□ Reproductive tract infection/sexually transmitted disease treatment

□ Psychological health care

□ Pregnant/prenatal care

□ Contraception climacteric hygiene

□ Others (please specify)

22. Which way do you most desire to get RH services-related information? ([multiple](javascript:;)-[choice](javascript:;), a maximum of three choices )

□ Medical staff

□ Internet

□ Wechat/Micro-blogs

□ Radio and television

□ Friends and family

□ Books/newspapers/magazines

□ Brochures
